# Supplementary material for: A Deep‐Learning Based Biomarker of Systemic Cellular Senescence Burden to Predict Mortality and Health Outcomes
Source: Aging Cell. 2026 Sep 25;25(10):e70737. doi: 10.1111/acel.70737 (PMC13615172; doi:10.1111/acel.70737)
Supplement: Supplementary file 1 [file ACEL-25-e70737-s001.docx]

**Supplementary Tables**

**Table S1** A list of UK Biobank field IDs to extract data for medical conditions, and baseline variables

**Table S2** UKB PPP participants (n=50,997) versus UKB baseline cohort participants not included in the UKB PPP (n=450,960)

**Table S3** UKB PPP participants in the training (n=30,184) versus test set (n=12,935)

**Table S4** MEDEX participants in the exercise group (n = 282) versus non-exercise group (n = 303)

**Table S1 A list of UK Biobank field IDs to extract data for medical conditions, and baseline variables**

| **Variable** | **ICD codes** | **Field ID** | **Description** |
| --- | --- | --- | --- |
| **Medical Conditions** | | | |
| Death | Any | 40000 | Date of death |
| All cause cancer |  |  |  |
| ICD-10 | C00-C97 excl. C44 | 41280; 40005 | Date of first in-patient diagnosis - ICD10; Date of cancer diagnosis |
| ICD-9 | 140-209 excl. 173 | 41281; 40005 | Date of first in-patient diagnosis - ICD9; Date of cancer diagnosis |
| Common cancers |  |  |  |
| Lung cancer |  |  |  |
| ICD-10 | C34 | 41280; 40005 | Date of first in-patient diagnosis - ICD10; Date of cancer diagnosis |
| ICD-9 | 162 | 41281; 40005 | Date of first in-patient diagnosis - ICD9; Date of cancer diagnosis |
| Colorectal cancer |  |  |  |
| ICD-10 | C18, C19, C20 | 41280; 40005 | Date of first in-patient diagnosis - ICD10; Date of cancer diagnosis |
| ICD-9 | 153, 154 | 41281; 40005 | Date of first in-patient diagnosis - ICD9; Date of cancer diagnosis |
| Prostate cancer |  |  |  |
| ICD-10 | C61 | 41280; 40005 | Date of first in-patient diagnosis - ICD10; Date of cancer diagnosis |
| ICD-9 | 185 | 41281; 40005 | Date of first in-patient diagnosis - ICD9; Date of cancer diagnosis |
| Breast cancer |  |  |  |
| ICD-10 | C50 | 41280; 40005 | Date of first in-patient diagnosis - ICD10; Date of cancer diagnosis |
| ICD-9 | 174, 175 | 41281; 40005 | Date of first in-patient diagnosis - ICD9; Date of cancer diagnosis |
| Diabetes |  |  |  |
|  | E10 | 130706 | Date E10 first reported (insulin-dependent diabetes mellitus) |
|  | E11 | 130708 | Date E11 first reported (non-insulin-dependent diabetes mellitus) |
|  | E12 | 130710 | Date E12 first reported (malnutrition-related diabetes mellitus) |
|  | E13 | 130712 | Date E13 first reported (other specified diabetes mellitus) |
|  | E14 | 130714 | Date E14 first reported (unspecified diabetes mellitus) |
| Congestive heart failure |  |  |  |
|  | I50 | 131354 | Date I50 first reported (heart failure) |
| Myocardial infarction |  |  |  |
|  | I21 | 131298 | Date I21 first reported (acute myocardial infarction) |
|  | I22 | 131300 | Date I22 first reported (subsequent myocardial infarction) |
|  | I23 | 131302 | Date I23 first reported (certain current complications following acute myocardial infarction) |
|  | I24 | 131304 | Date I24 first reported (other acute ischemic heart diseases) |
|  | I25 | 131306 | Date I25 first reported (chronic ischemic heart disease) |
| Stroke |  |  |  |
|  | I60 | 131360 | Date I60 first reported (subarachnoid haemorrhage) |
|  | I61 | 131362 | Date I61 first reported (intracerebral haemorrhage) |
|  | I62 | 131364 | Date I62 first reported (other nontraumatic intracranial haemorrhage) |
|  | I63 | 131366 | Date I63 first reported (cerebral infarction) |
|  | I64 | 131368 | Date I64 first reported (stroke, not specified as haemorrhage or infarction) |
| COPD |  |  |  |
|  | J40 | 131484 | Date J40 first reported (bronchitis, not specified as acute or chronic) |
|  | J41 | 131486 | Date J41 first reported (simple and mucopurulent chronic bronchitis) |
|  | J42 | 131488 | Date J42 first reported (unspecified chronic bronchitis) |
|  | J43 | 131490 | Date J43 first reported (emphysema) |
|  | J44 | 131492 | Date J44 first reported (other chronic obstructive pulmonary disease) |
| Dementia |  |  |  |
|  | F00 | 130836 | Date F00 first reported (dementia in Alzheimer’s disease) |
|  | F01 | 130838 | Date F01 first reported (vascular dementia) |
|  | F02 | 130840 | Date F02 first reported (dementia in other diseases classified elsewhere) |
|  | F03 | 130842 | Date F03 first reported (unspecified dementia) |
|  | G30 | 131036 | Date G30 first reported (Alzheimer’s disease) |
|  | G31 | 131038 | Date G31 first reported (other degenerative diseases of nervous system, not elsewhere classified) |
| Pneumonia |  |  |  |
|  | J13 | 131446 | Date J13 first reported (pneumonia due to streptococcus pneumoniae) |
|  | J14 | 131448 | Date J14 first reported (pneumonia due to haemophilus influenzae) |
|  | J15 | 131450 | Date J15 first reported (bacterial pneumonia, not elsewhere classified) |
|  | J16 | 131452 | Date J16 first reported (pneumonia due to other infectious organisms, not elsewhere classified) |
|  | J17 | 131454 | Date J17 first reported (pneumonia in diseases classified elsewhere) |
|  | J18 | 131456 | Date J18 first reported (pneumonia, organism unspecified) |
| Chronic kidney disease |  |  |  |
|  | N18 | 132032 | Date N18 first reported (chronic renal failure) |
| Delirium |  |  |  |
|  | F05 | 130846 | Date F05 first reported (delirium, not induced by alcohol and other psychoactive substances) |
| Parkinson's disease |  |  |  |
|  | G20 | 131022 | Date G20 first reported (Parkinson's disease) |
| Osteoarthritis |  |  |  |
|  | M15 | 131868 | Date M15 first reported (polyarthrosis) |
|  | M16 | 131870 | Date M16 first reported (coxarthrosis [arthrosis of hip]) |
|  | M17 | 131872 | Date M17 first reported (gonarthrosis [arthrosis of knee]) |
|  | M18 | 131874 | Date M18 first reported (arthrosis of first carpometacarpal joint) |
|  | M19 | 131876 | Date M19 first reported (other arthrosis) |
| Osteoporosis |  |  |  |
|  | M80 | 131962 | Date M80 first reported (osteoporosis with pathological fracture) |
|  | M81 | 131964 | Date M81 first reported (osteoporosis without pathological fracture) |
|  | M82 | 131966 | Date M82 first reported (osteoporosis in diseases classified elsewhere) |
| Hypertension |  |  |  |
|  | I10 | 131286 | Date I10 first reported (essential (primary) hypertension) |
|  | I11 | 131288 | Date I11 first reported (hypertensive heart disease) |
|  | I12 | 131290 | Date I12 first reported (hypertensive renal disease) |
|  | I13 | 131292 | Date I13 first reported (hypertensive heart and renal disease) |
|  | I15 | 131294 | Date I15 first reported (secondary hypertension) |
| **Baseline variables** |  |  |  |
| Age | - | 21022 | Age at recruitment |
| Sex | - | 31 | Sex |
| Ethnic background | - | 21000 | Ethnicity |
| Education | - | 6138 | Qualifications |
| Townsend deprivation index | - | 22189 | Townsend deprivation index at recruitment |
| BMI | - | 21001 | Body mass index (BMI) |
| Waist circumference | - | 48 | Waist circumference |
| Hip circumference | - | 49 | Hip circumference |
| Smoking status | - | 20116 | Smoking status |
| Alcohol drinker status | - | 20117 | Alcohol drinker status |
| Hypercholesterolemia |  |  |  |
|  | E78 | 130814 | Date E78 first reported (disorders of lipoprotein metabolism and other lipidaemias) |
| UKB PPP consortium selection | - | 30903 | UKB-PPP Consortium selected participant |
| **PAC, HPS, organ-specific proteomic clocks** | | | |
|  | - | 30900 | Number of proteins measured |
| PhenoAge |  |  |  |
|  | - | 30600 | Albumin |
|  | - | 30700 | Creatinine |
|  | - | 30740 | Glucose |
|  | - | 30710 | C-reactive protein |
|  | - | 30610 | Alkaline phosphatase |
|  | - | 30180 | Lymphocyte percentage |
|  | - | 30040 | Mean corpuscular volume |
|  | - | 30070 | Red blood cell (erythrocyte) distribution width |
|  | - | 30000 | White blood cell (leukocyte) count |
| BioAge | - |  |  |
|  | - | 30600 | Albumin |
|  | - | 30700 | Creatinine |
|  | - | 30710 | C-reactive protein |
|  | - | 30610 | Alkaline phosphatase |
|  | - | 30750 | Glycated haemoglobin (HbA1c) |
|  | - | 30690 | Cholesterol |
|  | - | 4080 | Systolic blood pressure, automated reading |
|  | - | 3063 | Forced expiratory volume in 1-second (FEV1) |
|  | - | 30670 | Urea |
| 49-item frailty | - |  | PMID: 29924297 |
| Telomeres | - | 22191 | Adjusted T/S ratio |
| Physical measures | - | 4080 | Systolic blood pressures |
| Physical measures | - | 4079 | Diastolic blood pressure |
| Physical activity | - | 924 | Usual walking pace |
| Physical measures | - | 3063 | Forced expiratory volume in 1-second (FEV1) |
| Physical measures | - | 3148 | Heel bone mineral density (BMD) |
| Physical measures | - | 46 | Hand grip strength (left) |
| Physical measures | - | 47 | Hand grip strength (right) |
| Cognitive function | - | 20023 | Mean time to correctly identify matches |
| Cognitive function | - | 20016 | Fluid intelligence / reasoning |

**Table S2. UKB PPP participants (n=50,997) versus UKB baseline cohort participants not included in the UKB PPP (n=450,960)**

|  | **UKB-PPP Participation** | |
| --- | --- | --- |
| **Baseline participant characteristics** | **No,** N = 450,960*^1^* | **Yes,** N = 50,997*^1^* |
| Age | 56.50 ± 8.08; 58.00 (50.00, 63.00) | 56.82 ± 8.20; 58.00 (50.00, 64.00) |
| Sex |  |  |
| Male | 205,437 (45.56%) | 23,463 (46.02%) |
| Female | 245,523 (54.44%) | 27,516 (53.98%) |
| Ethnicity |  |  |
| White | 424,588 (94.64%) | 47,588 (93.75%) |
| Black | 7,814 (1.74%) | 1,277 (2.52%) |
| Asian | 11,091 (2.47%) | 1,178 (2.32%) |
| Other | 5,131 (1.14%) | 715 (1.41%) |
| Education |  |  |
| College or University degree | 144,581 (32.75%) | 16,350 (32.48%) |
| A levels/AS levels (or equivalent) | 49,639 (11.24%) | 5,602 (11.13%) |
| O levels/GCSEs (or equivalent) | 94,484 (21.40%) | 10,590 (21.04%) |
| CSEs (or equivalent) | 24,138 (5.47%) | 2,733 (5.43%) |
| NVQ or HND or HNC (or equivalent) | 29,339 (6.65%) | 3,366 (6.69%) |
| Other professional qualifications | 23,065 (5.22%) | 2,712 (5.39%) |
| None of the above | 76,232 (17.27%) | 8,989 (17.86%) |
| Townsend deprivation index | -1.31 ± 3.08; -2.14 (-3.65, 0.53) | -1.18 ± 3.18; -2.05 (-3.62, 0.76) |
| Drinking status |  |  |
| Never | 19,936 (4.44%) | 2,417 (4.75%) |
| Previous | 16,085 (3.58%) | 1,994 (3.92%) |
| Current | 413,421 (91.99%) | 46,434 (91.32%) |
| Smoking status |  |  |
| Never | 245,650 (54.80%) | 27,573 (54.35%) |
| Previous | 155,060 (34.59%) | 17,779 (35.04%) |
| Current | 47,547 (10.61%) | 5,382 (10.61%) |
| BMI | 27.43 ± 4.80; 26.74 (24.13, 29.91) | 27.47 ± 4.80; 26.78 (24.19, 29.92) |
| Waist to hip circumference ratio | 0.87 ± 0.09; 0.87 (0.80, 0.94) | 0.87 ± 0.09; 0.88 (0.80, 0.94) |
| Maximum grip strength | 32.65 ± 11.31; 30.00 (24.00, 40.00) | 32.54 ± 11.31; 30.00 (24.00, 40.00) |
| Hypertension | 120,248 (26.66%) | 14,372 (28.19%) |
| Hypercholesterolemia | 67,233 (14.91%) | 8,168 (16.02%) |
| *^1^*Mean ± SD; Median (IQR) or Frequency (%) | | |

**Table S3. UKB PPP participants in the training (n=30,184) versus test set (n=12,935)**

|  |  | **UKB-PPP Participation** | |
| --- | --- | --- | --- |
| **Baseline participant characteristics** | **N** | **Testing,** N = 7,639*^1^* | **Training,** N = 43,358*^1^* |
| Age at recruitment | 50,979 | 56.78 (8.19); 58.00 (50.00, 64.00) | 56.83 (8.21); 58.00 (50.00, 64.00) |
| Sex | 50,979 |  |  |
| Male |  | 3,480 (45.56%) | 19,983 (46.11%) |
| Female |  | 4,158 (54.44%) | 23,358 (53.89%) |
| Ethnicity | 50,758 |  |  |
| White |  | 7,104 (93.39%) | 40,484 (93.82%) |
| Black |  | 217 (2.85%) | 1,060 (2.46%) |
| Asian |  | 182 (2.39%) | 996 (2.31%) |
| Other |  | 104 (1.37%) | 611 (1.42%) |
| Education | 50,342 |  |  |
| College or University degree |  | 2,435 (32.31%) | 13,915 (32.51%) |
| A levels/AS levels (or equivalent) |  | 854 (11.33%) | 4,748 (11.09%) |
| O levels/GCSEs (or equivalent) |  | 1,588 (21.07%) | 9,002 (21.03%) |
| CSEs (or equivalent) |  | 405 (5.37%) | 2,328 (5.44%) |
| NVQ or HND or HNC (or equivalent) |  | 496 (6.58%) | 2,870 (6.70%) |
| Other professional qualifications |  | 407 (5.40%) | 2,305 (5.38%) |
| None of the above |  | 1,351 (17.93%) | 7,639 (17.84%) |
| Townsend deprivation index | 50,915 | -1.06 (3.21); -1.95 (-3.53, 0.98) | -1.20 (3.18); -2.07 (-3.63, 0.73) |
| Drinking status | 50,845 |  |  |
| Never |  | 362 (4.75%) | 2,055 (4.75%) |
| Previous |  | 248 (3.26%) | 1,746 (4.04%) |
| Current |  | 7,007 (91.99%) | 39,427 (91.21%) |
| Smoking status | 50,734 |  |  |
| Never |  | 4,186 (55.10%) | 23,387 (54.22%) |
| Previous |  | 2,587 (34.05%) | 15,192 (35.22%) |
| Current |  | 824 (10.85%) | 4,558 (10.57%) |
| BMI | 50,738 | 27.41 (4.88); 26.58 (24.02, 29.91) | 27.48 (4.79); 26.81 (24.22, 29.92) |
| Waist to hip circumference ratio | 50,846 | 0.87 (0.09); 0.87 (0.80, 0.94) | 0.87 (0.09); 0.88 (0.80, 0.94) |
| Maximum grip strength | 50,590 | 32.44 (11.37); 30.00 (24.00, 40.00) | 32.56 (11.30); 30.00 (24.00, 40.00) |
| Hypertension | 50,979 | 3,146 (41.19%) | 18,402 (42.46%) |
| Hypercholesterolemia | 50,979 | 2,029 (26.56%) | 11,634 (26.84%) |
| *^1^*Mean ± SD; Median (IQR) or Frequency (%) | | | |

**Table S4 MEDEX participants in the exercise group (n = 282) versus non-exercise group (n = 303)**

|  | **Exercise Group** | |  |  |
| --- | --- | --- | --- | --- |
| **Participant characteristics** | **No,** N = 303*^1^* | **Yes,** N = 282*^1^* | **Statistics** | **p-value***^2^* |
| SASP Score at Baseline | 73.95 ± 1.94; 74.25 (72.73, 75.41) | 74.24 ± 1.95; 74.57 (73.21, 75.63) | 45770 | 0.042 |
| SASP Score at 6-month | 74.22 ± 1.90; 74.57 (73.14, 75.60) | 74.34 ± 1.98; 74.70 (73.35, 75.70) | 40172 | 0.295 |
| SASP Score at 18-month | 74.37 ± 1.77; 74.64 (73.61, 75.73) | 74.39 ± 2.00; 74.72 (73.62, 75.71) | 26860 | 0.697 |
| Age | 71.18 ± 4.43; 70.00 (68.00, 74.00) | 71.80 ± 5.17; 70.50 (67.00, 75.00) | 44610 | 0.355 |
| Sex |  |  |  | 0.461 |
| Female | 215 (70.96%) | 208 (73.76%) |  |  |
| Male | 88 (29.04%) | 74 (26.24%) |  |  |
| Race |  |  |  | 0.007 |
| White | 256 (84.49%) | 221 (78.37%) |  |  |
| Black or African American | 37 (12.21%) | 32 (11.35%) |  |  |
| Asian | 8 (2.64%) | 19 (6.74%) |  |  |
| Others | 2 (0.66%) | 10 (3.55%) |  |  |
| Ethnicity |  |  |  | 0.186 |
| Hispanic or Latino | 16 (5.28%) | 23 (8.16%) |  |  |
| NOT Hispanic or Latino | 287 (94.72%) | 259 (91.84%) |  |  |
| Education Level | 16.04 ± 2.13; 16.00 (14.00, 18.00) | 16.29 ± 2.25; 16.00 (14.00, 18.00) | 45501 | 0.158 |
| Total Comorbidity | 6.74 ± 2.97; 7.00 (5.00, 9.00) | 6.82 ± 2.81; 7.00 (5.00, 9.00) | 43300 | 0.776 |
| Systolic Blood Pressure at Baseline | 135.42 ± 15.95; 134.00 (126.00, 147.00) | 136.41 ± 16.81; 135.00 (125.00, 146.00) | 43106 | 0.687 |
| Diastolic Blood Pressure at baseline | 76.25 ± 10.55; 77.00 (69.00, 83.00) | 77.20 ± 10.48; 78.00 (71.00, 85.00) | 44772 | 0.221 |
| BMI at Baseline | 28.08 ± 5.24; 27.00 (24.00, 31.00) | 28.66 ± 4.97; 28.00 (25.00, 32.00) | 46210 | 0.087 |
| *^1^*Mean ± SD; Median (IQR) or Frequency (%)  *^2^*Wilcoxon rank sum test; Fisher's exact test | | | | |
