# Supplementary material for: A Deep‐Learning Based Biomarker of Systemic Cellular Senescence Burden to Predict Mortality and Health Outcomes
Source: Aging Cell. 2026 Sep 25;25(10):e70737. doi: 10.1111/acel.70737 (PMC13615172; doi:10.1111/acel.70737)
Supplement: Supplementary file 3 [file ACEL-25-e70737-s003.docx]

**Supplementary Figures**

Fig. S1 [UKB test samples (n=7,639)] SASP score Spearman correlations with chronological age, other biological age measures, physiological, cognitive, and frailty measures at baseline.

Fig. S2 [UKB test samples (n=7,639)] Partial Spearman correlations between SASP score and biological age measures, physiological, cognitive, and frailty measures at baseline, controlling for chronological age.

Fig. S3 [UKB test samples (n=7,639)] Rank of relative contribution of individual proteins to the SASP Score. Contributions evaluated by mean absolute SHAP value across all explained samples for each protein.

Fig. S4 [UKB test samples (n=7,639)] Hazard ratio of mortality comparing SASP score to individual SASP proteins at baseline. Adjusted covariates: age, sex, ethnicity, BMI, education, Townsend, smoking status, systolic blood pressure.

Fig. S5 [UKB test samples (n=7,639)] Hazard ratio of CKD comparing SASP score to individual SASP proteins at baseline. Adjusted covariates: age, sex, ethnicity, BMI, education, Townsend, smoking status, systolic blood pressure.

Fig. S6 [UKB test samples (n=7,639)] Hazard ratio of COPD comparing SASP score to individual SASP proteins at baseline. Adjusted covariates: age, sex, ethnicity, BMI, education, Townsend, smoking status, systolic blood pressure.

Fig. S7 [UKB test samples (n=7,639)] Hazard ratio of delirium comparing SASP score to individual SASP proteins at baseline. Adjusted covariates: age, sex, ethnicity, BMI, education, Townsend, smoking status, systolic blood pressure.

Fig. S8 [UKB test samples (n=7,639)] Hazard ratio of dementia comparing SASP score to individual SASP proteins at baseline. Adjusted covariates: age, sex, ethnicity, BMI, education, Townsend, smoking status, systolic blood pressure.

Fig. S9 [UKB test samples (n=7,639)] Hazard ratio of diabetes comparing SASP score to individual SASP proteins at baseline. Adjusted covariates: age, sex, ethnicity, BMI, education, Townsend, smoking status, systolic blood pressure.

Fig. S10 [UKB test samples (n=7,639)] Hazard ratio of heart failure comparing SASP score to individual SASP proteins at baseline. Adjusted covariates: age, sex, ethnicity, BMI, education, Townsend, smoking status, systolic blood pressure.

Fig. S11 [UKB test samples (n=7,639)] Hazard ratio of MI comparing SASP score to individual SASP proteins at baseline. Adjusted covariates: age, sex, ethnicity, BMI, education, Townsend, smoking status, systolic blood pressure.

Fig. S12 [UKB test samples (n=7,639)] Hazard ratio of stroke comparing SASP score to individual SASP proteins at baseline. Adjusted covariates: age, sex, ethnicity, BMI, education, Townsend, smoking status, systolic blood pressure.

Fig. S13 [UKB test samples (n=7,639)] Hazard ratio of all cause cancer comparing SASP score to individual SASP proteins at baseline. Adjusted covariates: age, sex, ethnicity, BMI, education, Townsend, smoking status, systolic blood pressure.

Fig. S14 [UKB test samples (n=7,639)] C-statistics for mortality predicted by baseline SASP score and individual proteins.

Fig. S15 [UKB test samples (n=7,639)] C-statistics for incident CKD predicted by baseline SASP score and individual proteins.

Fig. S16 [UKB test samples (n=7,639)] C-statistics for incident COPD predicted by baseline SASP score and individual proteins.

Fig. S17 [UKB test samples (n=7,639)] C-statistics for incident delirium predicted by baseline SASP score and individual proteins.

Fig. S18 [UKB test samples (n=7,639)] C-statistics for incident dementia predicted by baseline SASP score and individual proteins.

Fig. S19 [UKB test samples (n=7,639)] C-statistics for incident diabetes predicted by baseline SASP score and individual proteins.

Fig. S20 [UKB test samples (n=7,639)] C-statistics for incident heart failure predicted by baseline SASP score and individual proteins.

Fig. S21 [UKB test samples (n=7,639)] C-statistics for incident myocardial infarction predicted by baseline SASP score and individual proteins.

Fig. S22 [UKB test samples (n=7,639)] C-statistics for incident stroke predicted by baseline SASP score and individual proteins.

Fig. S23 [UKB test samples (n=7,639)] C-statistics for incident all cause cancer predicted by baseline SASP score and individual proteins.

Fig. S24 [MEDEX test samples (n=585)] Pearson’s correlation coefficients between SASP Score and baseline chronological age, BMI, Rockwood frailty, diastolic blood pressure, systolic blood pressure, leukocyte telomere length.

Fig. S25 [MEDEX test samples (n=585)] Partial Pearson’s correlation coefficients between SASP Score and BMI, Rockwood frailty, diastolic blood pressure, systolic blood pressure, leukocyte telomere length, controlling for baseline chronological age.

Fig. S26 [MEDEX (n=585)] Baseline SASP Score distribution comparing people with or without different medical conditions at baseline (a) high blood cholesterol, (b) high blood cholesterol, (c) chronic medical conditions (diabetes, kidney diseases, heart attack, heart disease) (d) previous or current smoker.

**Fig. S1** [UKB test samples (n=7,639)] SASP score Spearman correlations with chronological age, other biological age measures, physiological, cognitive, and frailty measures at baseline.

**Fig. S2** [UKB test samples (n=7,639)] Partial Spearman correlations between SASP score and biological age measures, physiological, cognitive, and frailty measures at baseline, controlling for chronological age.

**Fig. S3** [UKB test samples (n=7,639)] Rank of relative contribution of individual proteins to the SASP Score. Contributions evaluated by mean absolute SHAP value across all explained samples for each protein.

**Fig. S4** [UKB test samples (n=7,639)] Hazard ratio of mortality comparing SASP score to individual SASP proteins at baseline. Adjusted covariates: age, sex, ethnicity, BMI, education, Townsend, smoking status, systolic blood pressure.

**Fig. S5** [UKB test samples (n=7,639)] Hazard ratio of CKD comparing SASP score to individual SASP proteins at baseline. Adjusted covariates: age, sex, ethnicity, BMI, education, Townsend, smoking status, systolic blood pressure.

**Fig. S6** [UKB test samples (n=7,639)] Hazard ratio of COPD comparing SASP score to individual SASP proteins at baseline. Adjusted covariates: age, sex, ethnicity, BMI, education, Townsend, smoking status, systolic blood pressure.

**Fig. S7** [UKB test samples (n=7,639)] Hazard ratio of delirium comparing SASP score to individual SASP proteins at baseline. Adjusted covariates: age, sex, ethnicity, BMI, education, Townsend, smoking status, systolic blood pressure.

**Fig. S8** [UKB test samples (n=7,639)] Hazard ratio of dementia comparing SASP score to individual SASP proteins at baseline. Adjusted covariates: age, sex, ethnicity, BMI, education, Townsend, smoking status, systolic blood pressure.

**Fig. S9** [UKB test samples (n=7,639)] Hazard ratio of diabetes comparing SASP score to individual SASP proteins at baseline. Adjusted covariates: age, sex, ethnicity, BMI, education, Townsend, smoking status, systolic blood pressure.

**Fig. S10** [UKB test samples (n=7,639)] Hazard ratio of heart failure comparing SASP score to individual SASP proteins at baseline. Adjusted covariates: age, sex, ethnicity, BMI, education, Townsend, smoking status, systolic blood pressure.

**Fig. S11** [UKB test samples (n=7,639)] Hazard ratio of MI comparing SASP score to individual SASP proteins at baseline. Adjusted covariates: age, sex, ethnicity, BMI, education, Townsend, smoking status, systolic blood pressure.

**Fig. S12** [UKB test samples (n=7,639)] Hazard ratio of stroke comparing SASP score to individual SASP proteins at baseline. Adjusted covariates: age, sex, ethnicity, BMI, education, Townsend, smoking status, systolic blood pressure.

**Fig. S13** [UKB test samples (n=7,639)] Hazard ratio of all cause cancer comparing SASP score to individual SASP proteins at baseline. Adjusted covariates: age, sex, ethnicity, BMI, education, Townsend, smoking status, systolic blood pressure.

**Fig. S14** [UKB test samples (n=7,639)] C-statistics for mortality predicted by baseline SASP score and individual proteins.

**Fig. S15** [UKB test samples (n=7,639)] C-statistics for incident CKD predicted by baseline SASP score and individual proteins.

**Fig. S16** [UKB test samples (n=7,639)] C-statistics for incident COPD predicted by baseline SASP score and individual proteins.

**Fig. S17** [UKB test samples (n=7,639)] C-statistics for incident delirium predicted by baseline SASP score and individual proteins.

**Fig. S18** [UKB test samples (n=7,639)] C-statistics for incident dementia predicted by baseline SASP score and individual proteins.

**Fig. S19** [UKB test samples (n=7,639)] C-statistics for incident diabetes predicted by baseline SASP score and individual proteins.

**Fig. S20** [UKB test samples (n=7,639)] C-statistics for incident heart failure predicted by baseline SASP score and individual proteins.

**Fig. S21** [UKB test samples (n=7,639)] C-statistics for incident myocardial infarction predicted by baseline SASP score and individual proteins.

**Fig. S22** [UKB test samples (n=7,639)] C-statistics for incident stroke predicted by baseline SASP score and individual proteins.

**Fig. S23** [UKB test samples (n=7,639)] C-statistics for incident all cause cancer predicted by baseline SASP score and individual proteins.

**Fig. S24** [MEDEX test samples (n=585)] Spearman’s correlation coefficients between SASP Score and baseline chronological age, BMI, Rockwood frailty, diastolic blood pressure, systolic blood pressure, leukocyte telomere length.

**Fig. S25** [MEDEX test samples (n=585)] Partial Spearman’s correlation coefficients between SASP Score and BMI, Rockwood frailty, diastolic blood pressure, systolic blood pressure, leukocyte telomere length, controlling for baseline chronological age.

**Fig. S26** [MEDEX (n=585)] Baseline SASP Score distribution comparing people with or without different medical conditions at baseline (a) high blood cholesterol, (b) high blood cholesterol, (c) chronic medical conditions (diabetes, kidney diseases, heart attack, heart disease) (d) previous or current smoker.
